# Photo Quiz: Unexpected yeast in a premature infant—pathogen or passenger

**DOI:** 10.1128/jcm.01111-25

**Published:** 2025-10-08

**Authors:** Yuan Chao Xue, Lemuel O. Aigbivbalu, Katie J. Holekamp, Ping Ren

**Affiliations:** 1 Department of Pathology, University of Texas Medical Branch 198642, Galveston, Texas, USA; 2 Department of Pediatric Inpatient Services, University of Texas Medical Branch 12338, Galveston, Texas, USA; Mayo Clinic Minnesota, Rochester, Minnesota, USA

**Keywords:** lipid emulsion, neonate, *Malassezia pachydermatis*

## PHOTO QUIZ 

A 3-week-old male infant, born prematurely at 24 weeks’ gestation with extremely low birth weight, was admitted to the neonatal intensive care unit (NICU) for management of complications related to prematurity. Perinatal history was notable for a human immunodeficiency virus (HIV)-positive mother. Upon admission, the patient was intubated, placed on mechanical ventilation, and started on zidovudine for HIV prophylaxis.

On clinical evaluation, the infant exhibited apnea of prematurity (AOP) and a hemodynamically significant patent ductus arteriosus (PDA). AOP is characterized by intermittent cessation of breathing in preterm infants, while PDA refers to the persistence of a fetal blood vessel (ductus arteriosus) that can significantly alter postnatal circulation. Surgical ligation of the PDA was performed on hospital day (HD) 17. Postoperative imaging discovered pulmonary edema in the left lung, raising concern for impaired lymphatic drainage to the left pleural space.

The infant had been receiving parenteral nutrition, including SMOFlipid 20%, a lipid injectable emulsion, since birth due to gastrointestinal immaturity and feeding intolerance, common in extremely premature neonates. Initial HIV antibody testing was reactive, but an HIV nucleic acid amplification test (NAAT) obtained 5 days later was negative. NAATs for cytomegalovirus (CMV) in urine and for methicillin-resistant *Staphylococcus aureus* (MRSA)/methicillin-susceptible *S. aureus* (MSSA) from nasal swabs were also negative.

**Fig 1:**
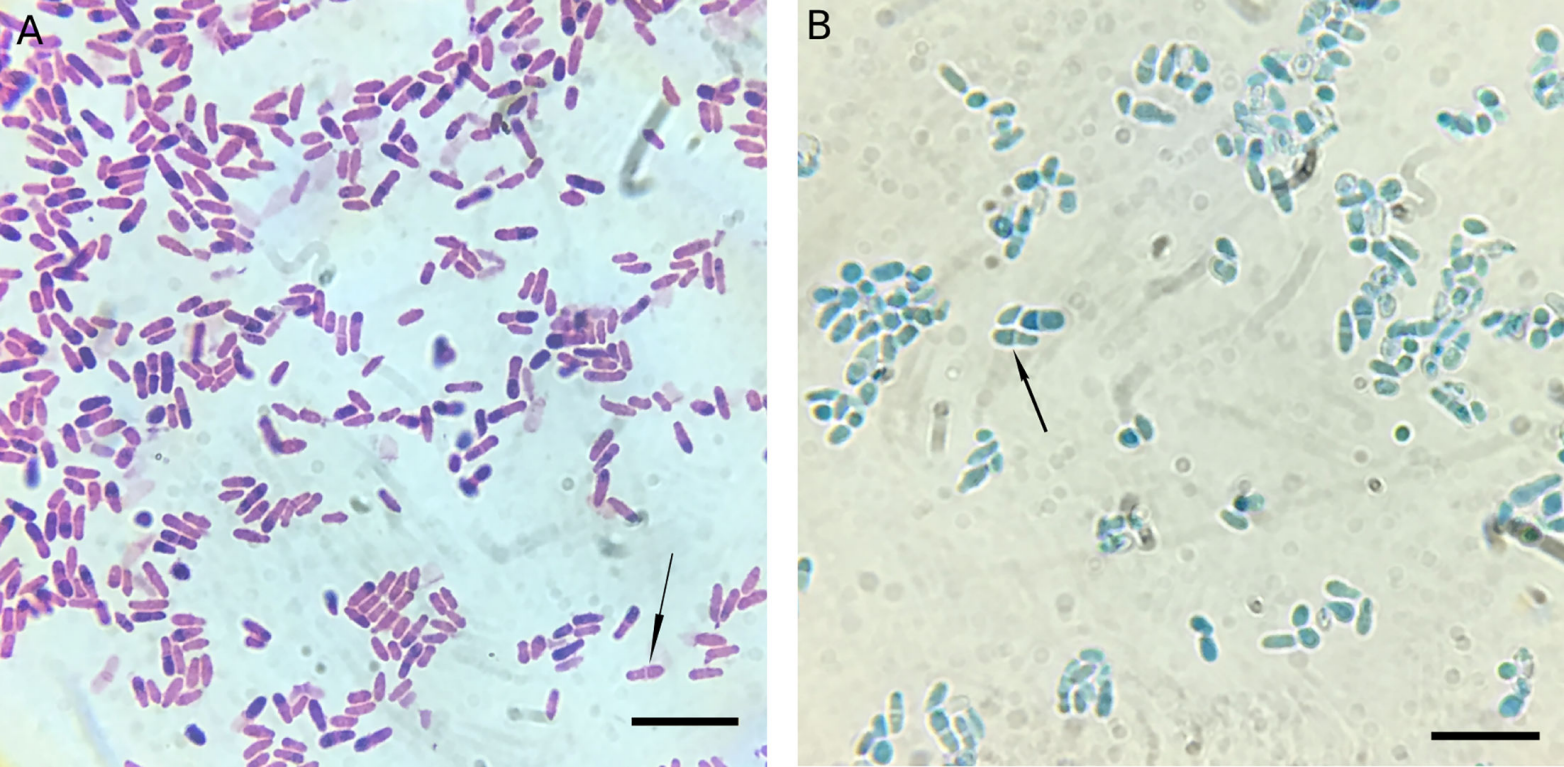
Gram stain (**A**) of the organism on chocolate agar and lactophenol cotton blue stain (**B**) of the organism on Sabouraud dextrose agar showing distinctive monopolar budding with “bowling pin” appearance (bar = 4 µm). Arrows indicate the collarettes.

Serial blood (every 2–6 days) and sputum (every 6 days) cultures collected since birth showed no clinically significant growth until HD 22. A sputum specimen collected on HD 20 was routinely cultured on sheep blood agar (SBA), chocolate agar (Choc), and MacConkey agar (MAC). Although no organisms were observed on the routine Gram stain of the sputum sample, pinpoint colonies (1+) appeared on both SBA and Choc but not on MAC after 48 h of incubation (36°C, 5% CO_2_). Gram staining of these colonies (Fig. 1A) revealed budding yeast, prompting subculture onto Sabouraud dextrose agar (SDA) (30°C, ambient air) for further characterization. Lactophenol cotton blue staining of the yeast from SDA is shown in Fig. 1B.

Repeating respiratory cultures, including bronchoalveolar lavage (BAL), tracheal aspirate, and sputum specimens collected over the following 4–10 days, were all negative for any pathogenic or predominant organisms. During this period, the only antimicrobial agent administered was ceftriaxone. No antifungal therapy was initiated because of the uncertain clinical significance of this organism in the respiratory tract.

What is your diagnosis?

## ANSWER TO THE PHOTO QUIZ

The organism isolated from the sputum culture was *Malassezia pachydermatis*, a yeast with uncertain clinical significance in the respiratory tract. In this case, the isolate likely represented colonization or contamination rather than true infection, serving more as a microbiological alarm than a clinically relevant pathogen.

Figure 1 shows numerous oval or peanut-shaped yeast cells with characteristic monopolar budding, producing a distinctive “bowling pin” appearance. Under high magnification, a collarette characterized as a small, collar-like lip at the mouth of a phialide can be clearly observed (Fig. 1). These microscopic features were consistent with *Malassezia* species. Notably, the organism grew on routine culture media without lipid supplementation, supporting identification as *M. pachydermatis*, the only non-lipid-dependent species in the genus. Identification was confirmed by matrix-assisted laser desorption/ionization time-of-flight mass spectrometry (MALDI-TOF MS) (Bruker MALDI Biotyper CA Version 3.2 and MALDI Biotyper CA library) without extraction.

*Malassezia* are lipophilic yeasts. Species in the *M. furfur* complex are primarily associated with human skin infections, such as pityriasis versicolor, whereas *M. pachydermatis* is more commonly found on the skin and mucosal surfaces of dogs and other animals (1, 2). Both groups can also colonize the skin of neonates shortly after birth, and cases of *Malassezia* fungemia have been reported in certain settings (3, 4). Human infections caused by *M. pachydermatis* are rare, although it is a well-known cause of otitis and dermatitis in animals (1). Nevertheless, *M. pachydermatis* has been implicated in sporadic cases and outbreaks of systemic infections in neonates, particularly in NICU settings (5, 6).

Risk factors for *Malassezia* infections in neonates include prematurity, low birth weight, central venous catheter use, and parenteral nutrition with lipid emulsions, which may promote yeast proliferation (6). In confirmed cases of invasive infections, management includes catheter removal, discontinuation of intravenous lipid emulsions, and targeted antifungal therapy (7).

Diagnosis can be suggested microscopically using stains such as KOH, lactophenol cotton blue, Gram, Giemsa, or Calcofluor white (8). Fungal culture remains essential, as lipid growth requirements help differentiate species. *M. pachydermatis* is distinguished by its ability to grow on standard media without lipid supplementation.

In summary, although *M. pachydermatis* is a known opportunistic pathogen in certain contexts, particularly in immunocompromised neonates receiving lipid-containing parenteral nutrition, its isolation here likely reflected colonization or contamination rather than infection, supported by negative follow-up cultures and clinical improvement without antifungal therapy. However, *M. pachydermatis* has been linked to NICU outbreaks, so even colonization may serve as a sentinel finding that warrants attention in vulnerable patient populations.
